# Plasma Fractionation Enriches Post-Myocardial Infarction Samples Prior to Proteomics Analysis

**DOI:** 10.1155/2012/397103

**Published:** 2012-06-18

**Authors:** Lisandra E. de Castro Brás, Kristine Y. DeLeon, Yonggang Ma, Qiuxia Dai, Kevin Hakala, Susan T. Weintraub, Merry L. Lindsey

**Affiliations:** ^1^San Antonio Cardiovascular Proteomics Center, The University of Texas Health Science Center at San Antonio, San Antonio, TX 78245, USA; ^2^Division of Geriatrics, Gerontology & Palliative Medicine, Department of Medicine, UTHSCSA, San Antonio, TX 78245, USA; ^3^Department of Biochemistry, UTHSCSA, San Antonio, TX 78245, USA

## Abstract

Following myocardial infarction (MI), matrix metalloproteinase-9 (MMP-9) levels increase, and MMP-9 deletion improves post-MI remodeling of the left ventricle (LV). We provide here a technical report on plasma-analysis from wild type (WT) and MMP-9 null mice using fractionation and mass-spectrometry-based proteomics. MI was induced by coronary artery ligation in male WT and MMP-9 null mice (4–8 months old; *n* = 3/genotype). Plasma was collected on days 0 (pre-) and 1 post-MI. Plasma proteins were fractionated and proteins in the lowest (fraction 1) and highest (fraction 12) molecular weight fractions were separated by 1-D SDS-PAGE, digested in-gel with trypsin and analyzed by HPLC-ESI-MS/MS on an Orbitrap Velos. We tried five different fractionation protocols, before reaching an optimized protocol that allowed us to identify over 100 proteins. Serum amyloid A substantially increased post-MI in both genotypes, while alpha-2 macroglobulin increased only in the null samples. In fraction 12, extracellular matrix proteins were observed only post-MI. Interestingly, fibronectin-1, a substrate of MMP-9, was identified at both day 0 and day 1 post-MI in the MMP-9 null mice but was only identified post-MI in the WT mice. In conclusion, plasma fractionation offers an improved depletion-free method to evaluate plasma changes following MI.

## 1. Introduction

Acute myocardial infarction (MI) remains a leading cause of morbidity and mortality worldwide. According to the latest report of the American Heart Association, every 25 seconds, an American will have a coronary event, and approximately every minute, someone will die of a coronary event [1]. In 2010, 785,000 Americans experienced an MI, and approximately 470,000 had a recurring MI [1]. Heart failure can result from adverse remodeling of the collagenous scar that replaces the damaged myocardium in the left ventricle (LV) after MI. LV remodeling is mediated by cell survival, inflammation, angiogenesis, and turnover of the extracellular matrix (ECM). Markers of LV remodeling can be either determined in the circulation (e.g., serum or plasma) or detected in the heart by imaging technologies or biopsy. Post-MI, levels of specific matrix metalloproteinases (MMPs) increase and mediate left ventricular remodeling. MMP-9 has been reported as a prognostic indicator of cardiac dysfunction in MI patients [2, 3]. MMP-9 deletion has also been shown to improve remodeling of the LV in mice [4, 5]. We hypothesized that the analysis of plasma proteins post-MI in wild-type (WT) and MMP-9 null mice will identify prospective markers of early MI that are MMP-9 dependent.

Termed as the most complex proteome, plasma is an intricate body fluid, containing a wide diversity of proteins [6]. Plasma has been investigated using targeted evaluations, to measure markers that detect MI or predict outcomes following MI. For examples, the muscle form of creatine kinase (CK-Mb), troponins, and C-reactive protein are used clinically to determine both presence of MI and extent of myocardial damage [7, 8]. MMP-9, galectin-3, and brain natriuretic peptide have been used to evaluate LV responses to MI [9–11]. Plasma has also been investigated using proteomic approaches, but this has been fraught with technical issues, primarily because the range of protein levels in the plasma is 10^10^, and the ten most abundant proteins account for 90% of the total protein concentration [12, 13]. Serum albumin is a high abundant protein in plasma, and it is the leading candidate for selective removal prior to proteomics analysis of less abundant proteins in plasma. Several albumin-depletion methods are commercially available, mainly based in immunoaffinity columns. Albumin can also be removed by ligand chromatography [14, 15], and isoelectric trapping [16]. Nonetheless, the use of depletion methods may also result in specific removal of low abundant cytokines, lipoproteins, and peptide hormones of interest [17]. 

Accordingly, we hypothesized that using a fractionation protocol for the analysis of plasma proteins post-MI in wild-type (WT) and MMP-9 null mice would identify prospective markers of early MI that are MMP-9 dependent. In our study, we performed protein fractionation prior to protein separation by 1D-PAGE and MS analysis. By doing so, we avoided using depletion methods and concomitantly reduced the presence of albumin and enriched for lower abundance proteins. 

## 2. Materials and Methods

### 2.1. Animals and Surgery

All animal procedures were conducted according to the ‘‘Guide for the Care and Use of Laboratory Animals” (NIH Notice Number: NOT-OD-12-020) and were approved by the Institutional Animal Care and Use Committee at the University of Texas at San Antonio. Male 4–8 months old C57BL6/J wild-type (WT) (*n* = 3) and MMP-9 null mice (*n* = 3) were used in this study. Animals were housed at constant temperature (22 ± 2°C) on a 12 h light/dark cycle. They were fed *ad libitum* on standard laboratory mice chow and had free access to tap water. MI was made by permanent ligation of the left anterior descending coronary artery as described previously [18]. Animals without MI (day 0) were used as controls (*n* = 3/genotype). At one day post-MI, mice were anesthetized with 5% isoflurane, plasma was collected, the coronary vasculature was flushed with 0.9 M saline, and the hearts were excised. The hearts were separated between right and left ventricles and were stained with 1% 2,3,5-triphenyltetrazolium chloride (Sigma) and photographed for measurement of infarct area.

### 2.2. Plasma Fractionation

Plasma was collected at days 0 and 1 post-MI, snap frozen and stored at −80°C. At sacrifice, heparin (100 *μ*L of 1000 USP Units/mL) was injected intraperitoneally, and 5 min after heparin injection, blood was collected from the carotid artery of the mouse. Total protein quantification was determined using Quick Start Bradford Protein Assay (Biorad). Plasma was fractionated using the GellFree 8100 Fractionation System (Protein Discovery, Inc.). Five hundred micrograms of total protein were reduced for 10 min at 50°C with 1x acetate sample buffer (Protein Discovery, Inc.) and 0.053 M dithiothreitol (DTT). After samples being cooled down to room temperature, 15 mM iodoacetamide was added, and samples were alkylated in the dark for 10 min. 

For protocol optimization, we used six different protocols where samples were either run in an 8%, 10%, or 12% Tris-acetate cartridge combined with one of three fractionation programs. We tested three different fractionation programs shown in Figures 2, 3, and 4. For all of the programs, MES was used as the running buffer (0.05 M MES, 0.05 M Tris, 0.1% SDS pH 7.9). For each of the six protocols tested, twelve fractions (150 *μ*L/fraction) were collected per sample and proteins were visualized on a 12% Bis-Tris gel by SDS-PAGE. 

### 2.3. Mass Spectrometry

The proteins in fraction 1 were separated in a 10–20% Tricine/peptide gel. The gel lane for each replicate was divided into six slices. The gel region containing visually detectable proteins from the lane for fraction 12 (the highest molecular weight fraction) on the Bis-Tris gel was excised into three slices. Each slice was separately destained and dehydrated and the proteins digested *in situ* with trypsin (Promega). The digests were analyzed by capillary HPLC-electrospray ionization tandem mass spectrometry (HPLC-ESI-MS/MS) on a Thermo Fisher LTQ Orbitrap Velos mass spectrometer fitted with a New Objective Digital PicoView 550 NanoESI source. Online HPLC separation of the digests was accomplished with an Eksigent/AB Sciex NanoLC-Ultra 2-D HPLC system: column, PicoFrit (New Objective; 75 *μ*m i.d.) packed to 15 cm with C18 adsorbent (Vydac; 218MS 5 *μ*m, 300 Å). Precursor ions were acquired in the Orbitrap in profile mode at 60,000 resolution (*m/z* 400); data-dependent collision-induced dissociation (CID) spectra of the six most intense ions in the precursor scan above a set threshold were acquired at the same time in the linear trap. Mascot (versions 2.3.02; Matrix Science) was used to search the uninterpreted CID spectra against a combination of the mouse subset of the NCBInr database (Mus. (145,083 sequences)) and a database of common contaminants (179 sequences). Methionine oxidation was considered as a variable modification; trypsin was specified as the proteolytic enzyme, with one missed cleavage allowed. A secondary search of the CID spectra using X! Tandem, cross-correlation of the X! Tandem and Mascot results, and determination of protein and peptide identity probabilities were accomplished by Scaffold (version 3; Proteome Software). The thresholds for acceptance of peptide and protein assignments in Scaffold were 95% and 99.9%, respectively. The results for the individual slices were combined for presentation purposes.

### 2.4. Immunoblotting

Proteins of interest were further analyzed by immunoblotting. Total proteins (10 *μ*g) were loaded onto either 4–12% Bis-Tris gels (proteins >50 kDa) or 10–20% tricine gels (proteins <50 kDa) and run by SDS-PAGE. Proteins were transferred to a nitrocellulose membrane which was hybridized overnight at 4°C with primary antibody. Primary antibodies used were antiserum amyloid A1 (number AF2948, R&D), anti-alpha-2 macroglobulin (number ab52651, Abcam) and antineutrophil-associated gelatinase lipocalin (NGAL, aka lipocalin 2; number ab63929, Abcam) (number ab63929, Abcam). Protein quantification was determined by densitometry analysis using ImageJ.

### 2.5. Statistical Analysis

Data are reported as mean ± SEM. Immunoblot intensities (arbitrary units) were assessed using a one-way ANOVA with Newman-Keuls multiple comparison test. A *P* < 0.05 was considered significant.

## 3. Results

**Figure 1:**
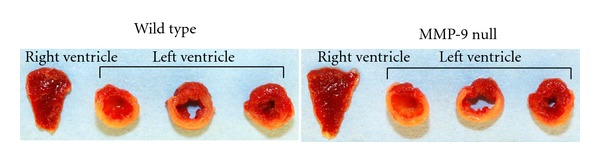
Infarct area was measured in the left ventricle. Infarct areas were similar between WT (52 ± 8%) and MMP-9 null (54 ± 2%) mice (*P* = 0.85).

**Figure 2:**
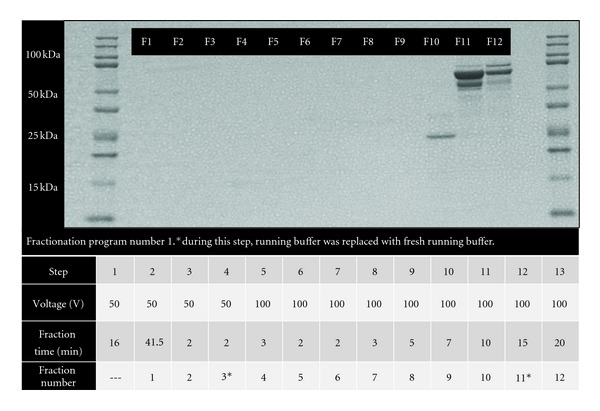
Plasma fractionation using an 8% acetate cartridge and program number 1; samples were run on 12% Bis-Tris gel. This fractionation scheme was not optimal because all of the proteins were observed in the last three fractions, rather than being evenly spread across fractions.

**Figure 3:**
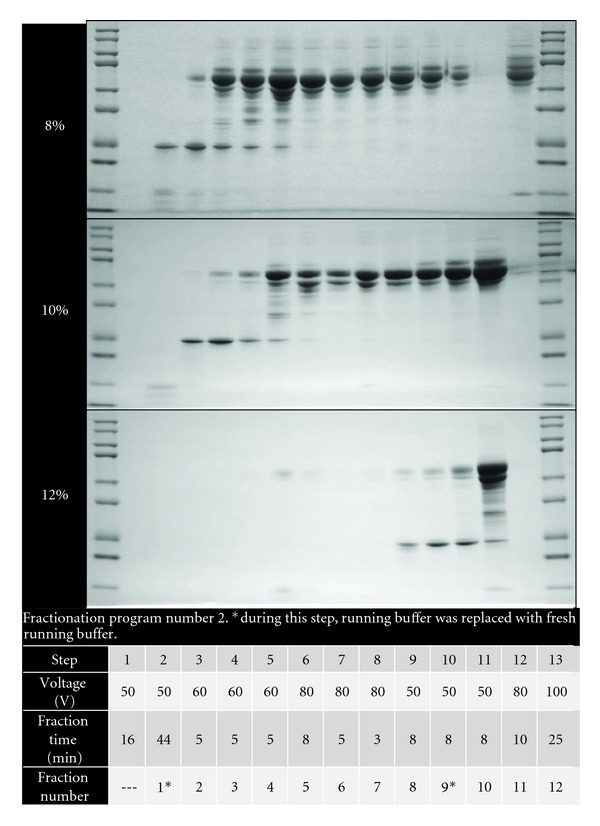
Three cartridges with different acetate percentages were used with the same fractionation program to study fraction protein profile. Program number 2 on 8%, 10% and 12% acetate cartridges and 12% Bis-Tris gels gave interesting results, in that the samples were spread out across fractions, but fraction 12 still showed high albumin abundance.

**Figure 4:**
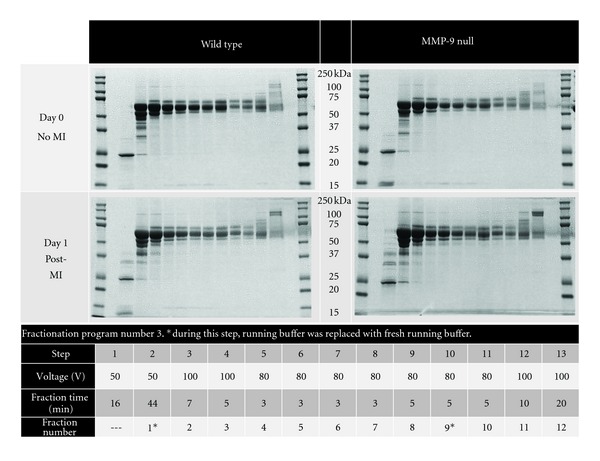
Each plasma sample was separated by electrophoretic mobility into 12 fractions, using fractionation program number 3 on an 8% acetate cartridge and were run on 12% Bis-Tris gels. The figure shows a representative gel from each group (*n* = 3 for each group).

Infarct areas were similar between WT and MMP-9 null mice (*P* = 0.85; Figure 1), indicating that both groups received a similar injury stimulus. We tested several methods to optimize the plasma fractionation prior to MS analysis. The different fractionation programs are shown in the figures. When using fractionation program number 1 and an 8% acetate cartridge, proteins were only observed in fractions 10 to 12 (Figure 2). By changing voltage intensities and step duration, we were able to visualize proteins in all 12 fractions. The protein' profiles differed depending of the type of cartridge used (Figure 3). Since serum albumin is approximately 66 kDa, we focused on protocols that provided fractions with reduced albumin content. The combination of program number 3 with the 8% acetate cartridge yielded fractions with these characteristics, where most of the albumin was seen in fractions 2 to 11 (Figure 4). These conditions were considered optimal for our examination, and fractions 1 and 12, which showed reduced levels of albumin, were further analyzed by HPLC-ESI-MS/MS on an Orbitrap Velos. 

**Figure 5:**
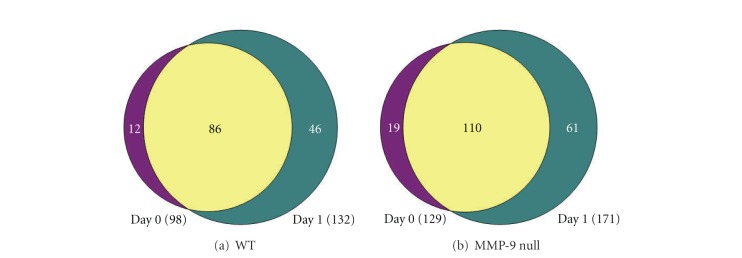
Venn diagram representing the number of proteins identified in combined fractions 1 and 12 of the plasma from each group. The purple is the number of proteins identified only in day 0. The green is the number of proteins identified at both time points. The Venn diagram was made using Venn Diagram Plotter software.

**Figure 6:**
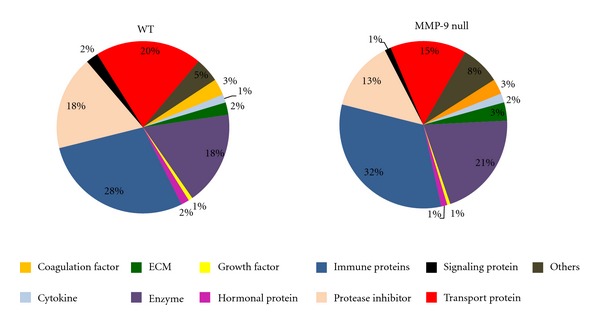
Proteins classification by biological function. The graph was created using the number of unweighted spectrum counts as a measure of relative abundance.

Supplemental Tables  1 and  2 list the proteins identified in both fractions (see Supplementary Material available online at doi:10.1155/2012/397103), per genotype and time point. Of the 145 proteins identified in the WT mice, 12 proteins were present only at day 0, and 45 proteins were just observed 1 day post-MI (Figure 5(a)). In the MMP-9 null mice, 195 proteins were identified; of which 19 were unique to day 0 and 61 proteins were observed only post-MI (Figure 5(b)). The molecular weight of proteins observed in fraction 1 ranged from 7 kDa to 69 kDa, although fragments of higher molecular weight proteins (e.g., C-terminus of alpha-2 macroglobulin) were also present. The majority of proteins observed in fraction 12 had molecular weights ranging from 45 kDa to 263 kDa; nevertheless, lower molecular weight proteins such as transthyretin (16 kDa) were also observed. The UniProt protein database was used to classify proteins by biological function. The unweighted spectrum counts were used to provide measure of relative abundance (Figure 6). Two percent of the proteins identified in WT animals were ECM proteins, while ECM proteins accounted for 3% of the total identified proteins in MMP-9 null mice.

**Figure 7:**
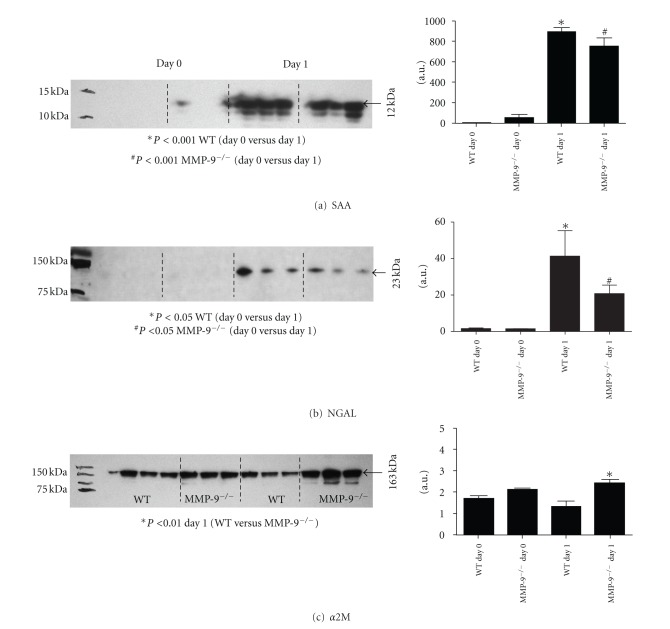
Immunoblots for: (a) Serum amyloid A. Protein levels increased significantly post-MI for both genotypes (*P* < 0.001). (b) NGAL. NGAL was observed only post-MI in both genotypes (*P* < 0.05). (c) Alpha-2 macroglobulin (*α*2M). MMP-9 null mice showed higher levels of *α*2M at 1 day post-MI compared to the WT mice counterpart. Densitometry, measured as arbitrary units (a.u.), was used to quantify protein levels in all immunoblots (*n* = 3/group).

We used immunoblotting as a secondary method to examine the proteins identified by MS. Serum amyloid A (SAA), a marker of inflammation, was observed at both time points. SAA was identified in fraction 1 of both genotypes and levels at day 0 were significantly different (*P* < 0.001) from levels at day 1 post-MI (Figure 7(a)). Eighty nine proteins were identified only post-MI, including NGAL. NGAL levels post-MI were significantly higher than at day 0 (*P* < 0.05) but no differences were observed between genotypes (Figure 7(b)). Alpha-2 macroglobulin, a generic MMP inhibitor and an MMP-9 substrate, was observed only post-MI in the WT group. Nevertheless, alpha-2 macroglobulin precursor was observed in both groups at days 0 and 1 post-MI. Quantification of alpha-2 macroglobulin showed a significant difference between genotypes 1 day post-MI (Figure 7(c)).

## 4. Discussion

The discovery of plasma markers remains challenging due to the complexity of the samples and the wide range of protein concentrations. In addition, the analysis of proteomics data is a complex multistep process [17]. Therefore, to overcome these problems, effective sample preparation is of outmost importance. Efficient sample preparation will reduce component complexity and enrich for lower abundance proteins while depleting or reducing the most abundant ones. We used a novel fractionation technique to interrogate plasma from WT and MMP-9 null mice at day 1 post-MI. This novel technique provides all of the advantages of 1-D gel electrophoresis, with the additional benefits of increased loading capacity and high yield liquid phase recovery. The most significant findings of this study were that (1) multiple proteins were identified in post-MI plasma, compared with day 0 control plasma; (2) serum amyloid A is good marker of early MI but it is not MMP-9 dependent; (3) alpha-2 macroglobulin may be an MMP-9 dependent marker. These results combined indicate that a fractionation followed by 1-D gel/LC/MS analysis strategy is effective to isolate and identify plasma proteins changes in response to MI.

We identified a total of 145 unique proteins in the WT samples and 195 unique proteins in the MMP-9 null samples. Known markers of inflammation, such as haptoglobin and SAA, were among the proteins identified. Studies from the Malmö Preventive Study, Sweden, have shown that elevated plasma levels of haptoglobin are a risk factor for MI [19]. Recently, Devaux's group identified haptoglobin as a potential biomarker of prognosis of heart failure in patients with acute MI [20]. Interestingly, they state that low levels of haptoglobin early post-MI favor heart failure. We identified haptoglobin as a potentially increased post-MI marker; nevertheless, studies with longer time points post-MI will have to be developed to confirm the role of haptoglobin in progression to heart failure. SAA is a known marker of inflammation, and SAA levels inversely correlate with cardiac function [21]. One-day post-MI the levels of SAA were significantly higher, in both genotypes, confirming an association with MI. Our future work involves a temporal study of plasma biomarkers post-MI. We plan to investigate if changes in plasma SAA are correlated with progression to heart failure post-MI.

From the proteins present only post-MI, we performed immunoblots against NGAL and alpha-2 macroglobulin. NGAL is a marker of kidney injury [22], as well as matrix degradation and inflammation [23]. This protein has previously been reported to be associated with MI and heart failure [24, 25]. A recent paper by Akcay and colleagues shows that 1-year mortality rates were significantly higher in patients with high levels of NGAL [26]. Our results were in accordance with the previous reports, showing a robust increase in NGAL levels post-MI. Alpha-2 macroglobulin is a generic proteinase inhibitor with broad specificity [27] and an MMP-9 substrate [28]. Although the association between MMP-9 and alpha-2 macroglobulin has been previously reported, this is the first time that alpha-2 macroglobulin is associated with MMP-9 in the myocardial infarction setting. The higher levels of alpha-2 macroglobulin observed in the MMP-9 mice post-MI suggest that this protein may be an MI biomarker that is MMP-9 dependent.

The protocols developed in this study can be used for other biological samples besides plasma. We are currently developing a fractionation method to investigate secreted proteins in cell culture media. Like in plasma, albumin is highly abundant in the commercially available serums used to supplement culture media. *In vitro*, the levels of secreted proteins are very low compared to the values observed in culture serum, making it very difficult to identify and quantify the proteins produced by the cells. Fractionation of samples is an easy and reproducible technique that can be used in a variety of models and biological samples.

Mouse models of MI are very useful and important given the unique ability to genetically manipulate these animals [29]. However, it is important to remember that the MI mouse model does not fully mimic the human disease. Thus, postinfarct remodeling of the LV likely has differences between the mouse and human that will need to be taken into account before full translation can occur. Acute MI remains a leading cause of morbidity and mortality worldwide. Thus, the discovery and development of biomarkers has high potential for providing a real benefit for screening, diagnosis, prognosis, prediction of recurrence, and therapeutic monitoring of MI patients.

In conclusion, by performing plasma fractionation prior to proteomics analysis, we were able to reduce the presence of high abundant proteins, such as albumin, and enrich samples for the detection of lower abundance proteins. We compared plasma samples from wild-type and MMP-9 null mice post-MI, and identified alpha-2 macroglobulin as a prospective MI marker which may be MMP-9 dependent. This technical report revealed that a fractionation approach is a useful technique to evaluate plasma samples.

## Supplementary Material

Proteins identified in the plasma of WT mice, listed alphabetically by group. Minimal requirements were kept at 99.9% probability for proteins, 95% probability for peptides and minimum of 2 unique peptides. Proteins identified in the MMP-9 null plasma samples, listed alphabetically by group. Minimal requirements were kept at 99.9% probability for proteins, 95% probability for peptides and minimum of 2 unique peptides.
