# Supplementary material for: Plasma Fractionation Enriches Post-Myocardial Infarction Samples Prior to Proteomics Analysis
Source: Int J Proteomics. 2012 Jun 18;2012:397103. doi: 10.1155/2012/397103 (PMC3385641; doi:10.1155/2012/397103)
Supplement: Supplementary file 1 — Proteins identified in the plasma of WT mice, listed alphabetically by group. Minimal requirements were kept at 99.9% probability for proteins, 95% probability for peptides and minimum of 2 unique peptides. Proteins identified in the MMP-9 null plasma samples, listed alphabetically by group. Minimal requirements were kept at 99.9% probability for proteins, 95% probability for peptides and minimum of 2 unique peptides. [file 397103.f1.docx]

| **Table 1.** Proteins identified in the plasma of WT mice, listed alphabetically by group. Minimal requirements were kept at 99.9% probability for proteins, 95% probability for peptides and minimum of 2 unique peptides. | | | | |
| --- | --- | --- | --- | --- |
| **#** | **Identified Proteins: WT day 0 only** | **Accession Number** | **Mol. Weight** | **Fraction** |
| **1** | apolipoprotein C-III, isoform CRA_b | gi\|148693736 (+2) | 7 kDa | 1 |
| **2** | coagulation factor XIII A chain precursor | gi\|30578393 (+3) | 83 kDa | 12 |
| **3** | complement C1q subcomponent subunit A | gi\|1168714 (+3) | 26 kDa | 1 |
| **4** | complement C1q subcomponent subunit C precursor | gi\|113680120 (+1) | 26 kDa | 1 |
| **5** | complement factor I precursor | gi\|110347406 (+2) | 67 kDa | 1 |
| **6** | extracellular superoxide dismutase | gi\|3320907 (+1) | 27 kDa | 1 |
| **7** | Fc fragment of IgG binding protein | gi\|169790797 (+2) | 275 kDa | 12 |
| **8** | Ig kappa light chain variable region | gi\|158346648 | 23 kDa | 1 |
| **9** | lymphoid-restricted membrane protein | gi\|187466274 (+7) | 56 kDa | 1 |
| **10** | phospholipid transfer protein precursor | gi\|6755112 | 54 kDa | 12 |
| **11** | proteasome subunit alpha type-3 | gi\|261824000 (+2) | 28 kDa | 1 |
| **12** | unnamed protein product | gi\|74203046 (+1) | 108 kDa | 1 |
| **#** | **Identified Proteins: WT day 0 and day 1 post-MI** | **Accession Number** | **Mol. Weight** | **Fraction** |
| **1** | afamin precursor | gi\|125347464 (+2) | 69 kDa | 12 |
| **2** | alpha-1 protease inhibitor 2 | gi\|191844 (+1) | 45 kDa | 12 |
| **3** | alpha-1-antitrypsin 1-3 | gi\|6678083 | 46 kDa | 12 |
| **4** | alpha-1-antitrypsin 1-4 precursor | gi\|6678085 | 46 kDa | 12 |
| **5** | alpha-1-antitrypsin 1-5 precursor | gi\|6678087 (+1) | 46 kDa | 12 |
| **6** | alpha-2-macroglobulin percursor | gi\|110347469(+2) | 166 kDa | 1 & 12 |
| **7** | apolipoprotein A-I preproprotein | gi\|160333304 (+1) | 31 kDa | 1 & 12 |
| **8** | apolipoprotein A-II | gi\|157057077 (+3) | 11 kDa | 1 |
| **9** | apolipoprotein E precursor | gi\|163644329 (+3) | 36 kDa | 1 |
| **10** | apolipoprotein M | gi\|18204576 (+1) | 21 kDa | 1 |
| **11** | apolipoprotein N | gi\|19527214 (+1) | 28 kDa | 1 |
| **12** | beta-globin | gi\|156257625 (+5) | 16 kDa | 1 |
| **13** | C4 complement protein | gi\|50242 | 127 kDa | 1 |
| **14** | C-reactive protein | gi\|117487 (+2) | 25 kDa | 12 |
| **15** | carboxylesterase | gi\|192854 (+1) | 61 kDa | 12 |
| **16** | carboxypeptidase N subunit 2 | gi\|147904569 (+3) | 60 kDa | 12 |
| **17** | ceruloplasmin, isoform CRA_a | gi\|148702936 (+3) | 124 kDa | 12 |
| **18** | chain A, crystal structure single chain trimer of MHC I heavy chain H-2kb | gi\|160285970 (+1) | 47 kDa | 1 |
| **19** | chain A, crystal structure of Fab-estradiol complexes | gi\|18655447 (+1) | 23 kDa | 1 |
| **20** | chain A, crystal structure of mouse transthyretin | gi\|161172183 | 14 kDa | 1 |
| **21** | chain A, S25-2 Fab unliganded 1 | gi\|42543426 | 24 kDa | 1 |
| **22** | chain L, anti-dinitrophenyl- spin-label monoclonal Ab Fab fragment | gi\|493856 | 24 kDa | 1 & 12 |
| **23** | chain L, crystal structure anti-arsonate germline Ab36-65 | gi\|110590782 (+4) | 24 kDa | 1 |
| **24** | chain L, crystal structure of the antiflavivirus Fab4g2 | gi\|62738079 | 23 kDa | 1 |
| **25** | chain L, Igg1 Fab fragment (of E8 Ab) complexed with horse cytochrome C | gi\|4139754 | 24 kDa | 1 |
| **26** | chain L, unliganded bactericidal Ab against Neisseria meningitidis | gi\|5107608 | 24 kDa | 1 |
| **27** | clusterin precursor | gi\|214010170 | 52 kDa | 1 & 12 |
| **28** | coagulation factor II | gi\|123227411 (+1) | 70 kDa | 12 |
| **29** | complement C1s-A subcomponent | gi\|148277054 (+4) | 77 kDa | 12 |
| **30** | complement C2 (within H-2S) | gi\|220897447 | 142 kDa | 12 |
| **31** | complement C3 | gi\|126518317 (+3) | 186 kDa | 1 & 12 |
| **32** | complement C4 | gi\|2944420 (+2) | 193 kDa | 12 |
| **33** | complement C5 preproprotein | gi\|6754164 | 189 kDa | 12 |
| **34** | complement C6 | gi\|161086891 (+1) | 87 kDa | 12 |
| **35** | complement C7 precursor | gi\|148671441 | 90 kDa | 12 |
| **36** | complement C8 gamma chain precursor | gi\|58037159 (+1) | 23 kDa | 1 |
| **37** | complement factor H | gi\|113926782 (+3) | 99 kDa | 12 |
| **38** | complement factor H-related protein C | gi\|110347406 (+2) | 67 kDa | 12 |
| **39** | epidermal growth factor receptor | gi\|10880776 (+2) | 135 kDa | 12 |
| **40** | fibrinogen, alpha polypeptide, isoform CRA_a | gi\|148683476 (+1) | 87 kDa | 1 |
| **41** | fibrinogen beta chain precursor | gi\|33859809 | 55 kDa | 12 |
| **42** | fibrinogen, gamma polypeptide | gi\|148683478 (+2) | 49 kDa | 12 |
| **43** | gelsolin precursor | gi\|28916693 | 86 kDa | 12 |
| **44** | glutathione peroxidase 3 isoform 1 | gi\|145275179 (+2) | 28 kDa | 1 |
| **45** | haptoglobin precursor | gi\|8850219 | 39 kDa | 1 & 12 |
| **46** | hemoglobin alpha, adult chain 2 | gi\|145301549 (+2) | 15 kDa | 1 |
| **47** | hemopexin precursor | gi\|160358829 (+1) | 51 kDa | 12 |
| **48** | histidine-rich glycoprotein | gi\|11066003 (+4) | 59 kDa | 12 |
| **49** | Ig gamma1 heavy chain | gi\|21304449 (+2) | 51 kDa | 12 |
| **50** | Ig gamma-2a chain | gi\|480895 | 52 kDa | 1 |
| **51** | Ig IgM kappa light chain | gi\|10121892 | 26 kDa | 1 |
| **52** | Ig joining chain | gi\|13543748 (+3) | 18 kDa | 1 |
| **53** | Ig kappa light chain | gi\|158264363 (+1) | 24 kDa | 1 |
| **54** | Ig kappa light chain | gi\|51921974 | 26 kDa | 1 |
| **55** | Ig mu chain C region (allele b) - mouse | gi\|90956 (+3) | 50 kDa | 12 |
| **56** | inhibitor of carbonic anhydrase | gi\|21313642 (+2) | 77 kDa | 12 |
| **57** | inter alpha-trypsin inhibitor, heavy chain 4 isoform 1 | gi\|226531047 (+2) | 105 kDa | 12 |
| **58** | inter-alpha trypsin inhibitor, heavy chain 2 | gi\|21707832 (+3) | 106 kDa | 12 |
| **59** | inter-alpha trypsin inhibitor, heavy chain 3 | gi\|148692826 (+1) | 99 kDa | 12 |
| **60** | inter-alpha-trypsin inhibitor heavy chain H1 precursor | gi\|124249351 | 101 kDa | 12 |
| **61** | kininogen-1 isoform 2 | gi\|12963497 | 48 kDa | 12 |
| **62** | leukemia inhibitory factor receptor, isoform CRA_b | gi\|148671402 (+2) | 82 kDa | 12 |
| **63** | lumican [Mus sp.] | gi\|1168179 (+2) | 38 kDa | 12 |
| **64** | macrophage colony-stimulating factor 1 receptor precursor | gi\|126723423 (+10) | 109 kDa | 12 |
| **65** | major urinary protein 14 | gi\|317008607 | 21 kDa | 1 |
| **66** | major urinary protein 5-like | gi\|149252566 (+1) | 21 kDa | 1 |
| **67** | mannan-binding lectin serine peptidase 1 | gi\|124297969 (+2) | 80 kDa | 12 |
| **68** | mannose-binding protein A precursor | gi\|6754654 | 25 kDa | 1 |
| **69** | mannose-binding protein C [Mus sp.] | gi\|233018 (+1) | 26 kDa | 1 |
| **70** | murinoglobulin-1 precursor | gi\|31982171 | 165 kDa | 12 |
| **71** | parvalbumin, isoform CRA_b | gi\|148697718 (+3) | 15 kDa | 1 |
| **72** | phosphatidylinositol-glycan-specific phospholipase D | gi\|111378397 (+2) | 94 kDa | 12 |
| **73** | plasma kallikrein precursor | gi\|236465805 (+2) | 71 kDa | 12 |
| **74** | plasma protease C1 inhibitor precursor | gi\|163914390 (+2) | 56 kDa | 12 |
| **75** | plasminogen | gi\|200403 (+2) | 91 kDa | 12 |
| **76** | proteasome subunit alpha type-7 | gi\|7106389 (+2) | 28 kDa | 1 |
| **77** | quiescin Q6, isoform CRA_b | gi\|148707454 (+2) | 84 kDa | 12 |
| **78** | retinol-binding protein | gi\|200679 (+3) | 20 kDa | 1 |
| **79** | serine (or cysteine) peptidase inhibitor, clade A, member 10, isoform CRA_a | gi\|148686875 (+2) | 55 kDa | 12 |
| **80** | serine (or cysteine) peptidase inhibitor, clade F, member 2, isoform CRA_b | gi\|148680863 (+1) | 56 kDa | 12 |
| **81** | serine protease inhibitor A3K precursor | gi\|148747546 | 47 kDa | 12 |
| **82** | serotransferrin precursor | gi\|20330802 | 77 kDa | 1 |
| **83** | serum albumin precursor | gi\|163310765 (+2) | 69 kDa | 1 & 12 |
| **84** | serum amyloid A | gi\|200904 (+1) | 13 kDa | 1 |
| **85** | serum amyloid A-4 protein precursor | gi\|6755398 | 15 kDa | 1 |
| **86** | serum amyloid P-component precursor | gi\|226958497 | 26 kDa | 1 |
| **87** | transferrin | gi\|17046471 (+1) | 77 kDa | 12 |
| **88** | vascular cell adhesion molecule 1, isoform CRA_a | gi\|148680441 (+4) | 81 kDa | 12 |
| **#** | **Identified Proteins: WT day 1 post-MI only** | **Accession Number** | **Mol. Weight** | **Fraction** |
| **1** | 14-3-3 zeta | gi\|1841387 | 28 kDa | 1 |
| **2** | alpha-1-microglobulin/bikunin precursor | gi\|311703 (+2) | 39 kDa | 1 |
| **3** | alpha-2-macroglobulin | gi\|199086 | 166 kDa | 1 & 12 |
| **4** | antithrombin-III precursor | gi\|18252782 | 52 kDa | 12 |
| **5** | apolipoprotein A-I preproprotein | gi\|160333304 (+1) | 31 kDa | 12 |
| **6** | apolipoprotein A-IV precursor | gi\|110347473 (+4) | 45 kDa | 1 & 12 |
| **7** | apolipoprotein B precursor | gi\|161702988 | 509 kDa | 12 |
| **8** | apolipoprotein E | gi\|192005 (+7) | 33 kDa | 12 |
| **9** | axonemal dynein heavy chain 8 long form | gi\|14335450 (+1) | 541 kDa | 1 |
| **10** | carbonic anhydrase 1 | gi\|116063531 (+1) | 28 kDa | 1 |
| **11** | carbonic anhydrase 2 | gi\|157951596 (+1) | 29 kDa | 1 |
| **12** | chain A, serpina3n, a murine orthologue of human antichymotrypsin | gi\|75765652 | 45 kDa | 12 |
| **13** | chain L, crystal structure of Fab fragment of mouse anti-human Fas Ab Hfe7a | gi\|18655521 | 24 kDa | 1 |
| **14** | claspin homolog (Xenopus laevis), isoform CRA_a | gi\|148698335 (+3) | 148 kDa | 1 |
| **15** | complement factor I precursor | gi\|110347406 (+2) | 67 kDa | 12 |
| **16** | cytochrome c, somatic | gi\|6681095 (+2) | 12 kDa | 1 |
| **17** | expressed in non-metastatic cells 2 protein | gi\|154550673 (+1) | 16 kDa | 1 |
| **18** | fetuin | gi\|2546995 (+2) | 37 kDa | 12 |
| **19** | fibronectin 1, isoform CRA_b | gi\|148667849 (+10) | 263 kDa | 12 |
| **20** | glycogen phosphorylase, muscle form | gi\|6755256 | 97 kDa | 12 |
| **21** | H2-Q10 protein | gi\|15029964 (+3) | 37 kDa | 1 |
| **22** | heat shock 70 kDa protein 4 | gi\|112293266 (+4) | 94 kDa | 12 |
| **23** | hepatocyte growth factor activator | gi\|18044173 (+3) | 71 kDa | 12 |
| **24** | Ig heavy chain [Mus sp.] | gi\|1794157 | 50 kDa | 12 |
| **25** | Ig light chain monoclonal antibody IgG4 | gi\|149799206 (+12) | 24 kDa | 12 |
| **26** | Igh protein | gi\|62028521 | 52kDa | 12 |
| **27** | Ighg protein | gi\|62024579 | 52kDa | 12 |
| **28** | LDHa protein | gi\|13529599 (+5) | 35 kDa | 1 |
| **29** | L-lactate dehydrogenase B chain | gi\|6678674 | 37 kDa | 1 |
| **30** | malate dehydrogenase 1, NAD (soluble), isoform CRA_c | gi\|148675904 (+2) | 40 kDa | 1 |
| **31** | microtubule-actin crosslinking factor 1 | gi\|123244271 (+2) | 832 kDa | 12 |
| **32** | neutrophil gelatinase-associated lipocalin precursor | gi\|34328049 (+1) | 23 kDa | 1 |
| **33** | phosphoglycerate mutase 2 | gi\|9256624 | 29 kDa | 1 |
| **34** | proteasome (prosome, macropain) subunit, beta type 6 | gi\|15530232 (+4) | 22 kDa | 1 |
| **35** | proteasome subunit alpha type-4 | gi\|6755196 (+2) | 29 kDa | 1 |
| **36** | protein S100-A9 | gi\|6677837 | 13 kDa | 1 |
| **37** | serine (or cysteine) peptidase inhibitor, clade A, member 1C | gi\|14602605 (+4) | 46 kDa | 1 |
| **38** | serine protease inhibitor A3M precursor | gi\|148686846 | 47 kDa | 12 |
| **39** | serine protease inhibitor A3N precursor | gi\|130503301 | 47 kDa | 12 |
| **40** | serum amyloid A (AA at 131) | gi\|200915 (+2) | 10 kDa | 1 |
| **41** | serum paraoxonase | gi\|1272236 (+3) | 40 kDa | 1 |
| **42** | transthyretin | gi\|7305599 | 16 kDa | 12 |
| **43** | triosephosphate isomerase | gi\|1864018 (+7) | 23 kDa | 1 |
| **44** | vinculin, isoform CRA_b | gi\|148669535 (+2) | 124 kDa | 12 |
| **45** | vitronectin | gi\|202372 | 55 kDa | 12 |
