# Supplementary material for: Plasma Fractionation Enriches Post-Myocardial Infarction Samples Prior to Proteomics Analysis
Source: Int J Proteomics. 2012 Jun 18;2012:397103. doi: 10.1155/2012/397103 (PMC3385641; doi:10.1155/2012/397103)
Supplement: Supplementary file 2 [file 397103.f2.docx]

| **Table 2.** Proteins identified in the MMP-9 null plasma samples, listed alphabetically by group**.** Minimal requirements were kept at 99.9% probability for proteins, 95% probability for peptides and minimum of 2 unique peptides. | | | | |
| --- | --- | --- | --- | --- |
| **#** | **Identified Proteins: MMP-9 null day 0 only** | **Accession Number** | **Mol. Weight** | **Fraction** |
| **1** | adenylate kinase isoenzyme 1 isoform 1 | gi\|10946936 (+1) | 23 kDa | 1 |
| **2** | antithrombin-III precursor | gi\|18252782 | 52 kDa | 12 |
| **3** | apolipoprotein C-III precursor | gi\|15421856 | 11 kDa | 1 |
| **4** | apolipoprotein D | gi\|575657 (+1) | 22 kDa | 1 |
| **5** | ATP-dependent RNA helicase Dhx29 | gi\|46852276 (+1) | 154 kDa | 12 |
| **6** | beta-globin | gi\|156257637 | 16 kDa | 1 |
| **7** | coagulation factor XIII A chain precursor | gi\|12836189 | 44 kDa | 12 |
| **8** | Col7a1 protein | gi\|38181520 | 52 kDa | 1 |
| **9** | collagen pro-alpha-1 type I chain | gi\|470674 | 138 kDa | 1 |
| **10** | Fc fragment of IgG binding protein | gi\|169790797 (+3) | 275 kDa | 12 |
| **11** | flavin reductase | gi\|21450325 | 22 kDa | 1 |
| **12** | heparin cofactor 2 precursor | gi\|161377465 (+1) | 54 kDa | 12 |
| **13** | murinoglobulin-1 precursor | gi\|31982171 (+2) | 165 kDa | 1 |
| **14** | myoglobin | gi\|21359820 | 17 kDa | 1 |
| **15** | peroxiredoxin 1 | gi\|123230136 (+4) | 19 kDa | 1 |
| **16** | Pf4 protein | gi\|38174332 (+2) | 11 kDa | 1 |
| **17** | phospholipid transfer protein precursor | gi\|6755112 | 54 kDa | 12 |
| **18** | titin | gi\|160358754 | 3906 kDa | 1 |
| **19** | thrombospondin 1, isoform CRA_b | gi\|148695955 (+6) | 127 kDa | 1 |
| **#** | **Identified Proteins: MMP-9 null day 0 and day 1 post-MI** | **Accession Number** | **Mol. Weight** | **Fraction** |
| **1** | agglutinating monoclonal antibody light chain | gi\|2950241 | 24 kDa | 1 |
| **2** | afamin precursor | gi\|125347464 (+1) | 69 kDa | 12 |
| **3** | alpha-1-antitrypsin 1-4 precursor | gi\|6678085 | 46 kDa | 1 & 12 |
| **4** | alpha-1-antitrypsin 1-5 precursor | gi\|6678087 (+1) | 46 kDa | 1 & 12 |
| **5** | alpha-1 protease inhibitor 2 | gi\|191844 (+1) | 45 kDa | 1 & 12 |
| **6** | alpha-2-macroglobulin precursor | gi\|110347469 | 166 kDa | 1 & 12 |
| **7** | anti-PRSV monoclonal ab PRSV-H 10-9 Ig heavy chain | gi\|50346336 | 51 kDa | 12 |
| **8** | alpha-1-antitrypsin 1-5 precursor | gi\|6678087 (+1) | 46 kDa | 12 |
| **9** | alpha-1 protease inhibitor 2 | gi\|191844 (+1) | 45 kDa | 12 |
| **10** | apolipoprotein A-I, isoform CRA_b | gi\|148693731 (+3) | 29 kDa | 12 |
| **11** | apolipoprotein A-I preproprotein | gi\|160333304 (+1) | 31 kDa | 1 |
| **12** | apolipoprotein A-II | gi\|157057077 (+3) | 11 kDa | 1 |
| **13** | apolipoprotein A-IV precursor | gi\|110347473 (+2) | 45 kDa | 1 |
| **14** | apolipoprotein B precursor | gi\|161702988 | 509 kDa | 12 |
| **15** | apolipoprotein E precursor | gi\|74178217 | 36 kDa | 1 |
| **16** | apolipoprotein M | gi\|18204576 (+2) | 21 kDa | 1 |
| **17** | apolipoprotein N precursor | gi\|26350411 | 25 kDa | 1 |
| **18** | carbonic anhydrase 1 | gi\|116063531 | 28 kDa | 1 |
| **19** | carbonic anhydrase 2 | gi\|157951596 | 29 kDa | 1 |
| **20** | carboxylesterase | gi\|192854 (+1) | 61 kDa | 12 |
| **21** | carboxypeptidase N subunit 2 | gi\|147904569 (+3) | 60 kDa | 12 |
| **22** | ceruloplasmin, isoform CRA_a | gi\|148702936 (+3) | 124 kDa | 12 |
| **23** | chain A, crystal structure single chain trimer of MHC I heavy chain H-2kb | gi\|160285970 (+1) | 47 kDa | 1 |
| **24** | chain A, crystal structure of mouse transthyretin | gi\|161172183 | 14 kDa | 1 & 12 |
| **25** | chain A, idiotope-anti-idiotope Fab-Fab complex | gi\|4930001 | 24 kDa | 1 |
| **26** | chain B, crystal structure of Dsbb-Fab complex | gi\|226887667 | 26 kDa | 1 |
| **27** | chain C, crystal structure complex of antibody & allergen Bla G 2 | gi\|168177050 | 23 kDa | 1 |
| **28** | chain L, complex (antibody/antigen) | gi\|3114497 | 24 kDa | 1 |
| **29** | chain L, crystal structure & paratope determination of 82d6a3 | gi\|83753979 | 23 kDa | 1 |
| **30** | chain L, crystal structure of complex Fab F9.13.7 & guinea-fowl lysozyme | gi\|999690 | 24 kDa | 1 |
| **31** | chain L, Fab fragment of Ab against epitope of Gp41 from Hiv-1 | gi\|1942810 (+3) | 24 kDa | 1 |
| **32** | clusterin precursor | gi\|214010170 (+1) | 52 kDa | 1 & 12 |
| **33** | coagulation factor II | gi\|123227411 (+1) | 70 kDa | 12 |
| **34** | complement C1q subcomponent subunit A | gi\|1168714 (+2) | 26 kDa | 1 |
| **35** | complement C1q subcomponent subunit B | gi\|115202 (+2) | 27 kDa | 1 |
| **36** | complement C1q subcomponent subunit C precursor | gi\|113680120 (+1) | 26 kDa | 1 |
| **37** | complement C1s-A subcomponent | gi\|148277054 (+5) | 77 kDa | 12 |
| **38** | complement C2 (within H-2S) | gi\|220897447 | 142 kDa | 12 |
| **39** | complement component 2 (within H-2S), isoform CRA_b | gi\|148694788 (+3) | 84 kDa | 12 |
| **40** | complement C3 | gi\|126518317 (+2) | 186 kDa | 1 & 12 |
| **41** | complement C4-B | gi\|126302537 (+2) | 193 kDa | 1 |
| **42** | complement C4-B precursor | gi\|157951698 (+1) | 193 kDa | 12 |
| **43** | complement C5 preproprotein | gi\|6754164 | 189 kDa | 12 |
| **44** | complement C6 | gi\|161086891 (+1) | 87 kDa | 12 |
| **45** | complement C7 precursor | gi\|148671441 | 90 kDa | 12 |
| **46** | complement C8 gamma chain precursor | gi\|58037159 | 23 kDa | 1 |
| **47** | complement factor I precursor | gi\|110347406 (+3) | 67 kDa | 1 |
| **48** | complement factor H | gi\|109627652 (+2) | 141 kDa | 12 |
| **49** | complement factor H-related protein C | gi\|113926782 (+3) | 99 kDa | 12 |
| **50** | C-reactive protein precursor | gi\|162138926 (+1) | 25 kDa | 1 |
| **51** | Cu/Zn superoxide dismutase | gi\|226471 (+1) | 16 kDa | 1 |
| **52** | epidermal growth factor receptor | gi\|10880776 (+5) | 135 kDa | 12 |
| **53** | fetuin | gi\|2546995 (+3) | 37 kDa | 1 & 12 |
| **54** | fibrinogen, alpha polypeptide, isoform CRA_a | gi\|148683476 (+1) | 87 kDa | 1 & 12 |
| **55** | fibrinogen beta chain precursor | gi\|33859809 | 55 kDa | 12 |
| **56** | fibrinogen, gamma polypeptide | gi\|148683478 (+2) | 49 kDa | 1 & 12 |
| **57** | fibronectin 1, isoform CRA_b | gi\|148667849 (+7) | 263 kDa | 12 |
| **58** | fructose-bisphosphate aldolase A isoform 2 | gi\|6671539 (+1) | 39 kDa | 1 |
| **59** | gelsolin precursor | gi\|28916693 | 86 kDa | 12 |
| **60** | glutathione peroxidase 3 isoform 1 | gi\|145275179 (+1) | 28 kDa | 1 |
| **61** | glyceraldehyde-3-phosphate dehydrogenase | gi\|55153885 (+6) | 36 kDa | 1 |
| **62** | haptoglobin precursor | gi\|8850219 | 39 kDa | 1 & 12 |
| **63** | hemoglobin alpha, adult chain 2 | gi\|145301549 (+2) | 15 kDa | 1 |
| **64** | hemoglobin subunit beta-1 | gi\|31982300 | 16 kDa | 1 |
| **65** | hemopexin | gi\|1881768 | 51 kDa | 1 & 12 |
| **66** | histidine-rich glycoprotein | gi\|11066003 (+4) | 59 kDa | 12 |
| **67** | Ig mu chain C region (allele b) - mouse | gi\|90956 (+3) | 50 kDa | 12 |
| **68** | Igh protein | gi\|62028521 | 52 kDa | 1 & 12 |
| **69** | Ighg protein | gi\|62024579 | 52 kDa | 12 |
| **70** | Igk protein | gi\|74215086 | 26 kDa | 1 |
| **71** | Ig joining chain | gi\|13543748 (+3) | 18 kDa | 1 |
| **72** | Ig lambda chain | gi\|37221149 | 23 kDa | 1 |
| **73** | Ig lambda chain | gi\|37196444 | 24 kDa | 1 |
| **74** | inhibitor of carbonic anhydrase | gi\|21313642 (+2) | 77 kDa | 12 |
| **75** | insulin-like growth factor binding protein | gi\|148690425 (+4) | 74 kDa | 12 |
| **76** | inter-alpha trypsin inhibitor, heavy chain 2 | gi\|21707832 (+3) | 106 kDa | 12 |
| **77** | inter-alpha trypsin inhibitor, heavy chain 3 | gi\|148692826 (+1) | 99 kDa | 12 |
| **78** | inter alpha-trypsin inhibitor, heavy chain 4 isoform 1 | gi\|226531047 (+2) | 105 kDa | 12 |
| **79** | inter-alpha-trypsin inhibitor heavy chain H1 precursor | gi\|124249351 | 101 kDa | 12 |
| **80** | kininogen-1 isoform 1 | gi\|156231021 (+4) | 73 kDa | 12 |
| **81** | leukemia inhibitory factor receptor isoform 1 precursor | gi\|7305235 (+1) | 123 kDa | 12 |
| **82** | lumican [Mus sp.] | gi\|1168179 (+2) | 38 kDa | 12 |
| **83** | macrophage colony-stimulating factor 1 receptor precursor | gi\|126723423 (+10) | 109 kDa | 12 |
| **84** | major urinary protein 14 | gi\|317008607 | 21 kDa | 1 |
| **85** | major urinary protein 5-like | gi\|149252566 (+1) | 21 kDa | 1 |
| **86** | mannan-binding lectin serine peptidase 1 | gi\|124297969 (+2) | 80 kDa | 12 |
| **87** | mannose-binding protein A precursor | gi\|6754654 | 25 kDa | 1 |
| **88** | mannose-binding protein C [Mus sp.] | gi\|233018 (+1) | 26 kDa | 1 |
| **89** | murinoglobulin-1 precursor | gi\|31982171 | 165 kDa | 1 & 12 |
| **90** | nucleoside diphosphate kinase B | gi\|6679078 (+1) | 17 kDa | 1 |
| **91** | parvalbumin, isoform CRA_b | gi\|148697718 (+3) | 15 kDa | 1 |
| **92** | peroxiredoxin-2 | gi\|148747558 (+2) | 22 kDa | 1 |
| **93** | phosphatidylinositol-glycan-specific phospholipase D | gi\|111378397 (+2) | 94 kDa | 12 |
| **94** | phosphoglycerate mutase 2 | gi\|9256624 | 29 kDa | 1 |
| **95** | plasma kallikrein precursor | gi\|236465805 (+2) | 71 kDa | 12 |
| **96** | plasma protease C1 inhibitor precursor | gi\|163914390 (+2) | 56 kDa | 12 |
| **97** | plasminogen | gi\|200403 (+2) | 91 kDa | 12 |
| **98** | proteasome (prosome, macropain) subunit, beta type 6 | gi\|15530232 (+3) | 22 kDa | 1 |
| **99** | proteasome subunit alpha type-7 | gi\|7106389 (+2) | 28 kDa | 1 |
| **100** | protein AMBP precursor | gi\|6680684 (+1) | 39 kDa | 1 |
| **101** | Q10 | gi\|387442 (+1) | 37 kDa | 1 |
| **102** | retinol-binding protein 4 isoform 1 | gi\|226958688 | 28 kDa | 1 |
| **103** | serine (or cysteine) peptidase inhibitor, clade A, member 10 | gi\|148686875 (+1) | 55 kDa | 12 |
| **104** | Serine (or cysteine) peptidase inhibitor, clade A, member 1C | gi\|14602605 (+1) | 46 kDa | 12 |
| **105** | serine (or cysteine) peptidase inhibitor, clade F, member 2 | gi\|148680863 (+1) | 56 kDa | 12 |
| **106** | serine protease inhibitor A3K precursor | gi\|148747546 | 47 kDa | 12 |
| **107** | serum albumin precursor | gi\|163310765 (+2) | 69 kDa | 1 & 12 |
| **108** | serum amyloid A | gi\|200904 (+1) | 13 kDa | 1 |
| **109** | serum amyloid A-4 protein precursor | gi\|6755398 | 15 kDa | 1 |
| **110** | serum amyloid P-component precursor | gi\|226958497 | 26 kDa | 1 |
| **111** | serum paraoxonase | gi\|1272236 (+3) | 40 kDa | 1 |
| **112** | sulfhydryl oxidase 1 isoform b | gi\|12963609 (+3) | 63 kDa | 12 |
| **113** | transferrin | gi\|17046471 (+2) | 77 kDa | 1 & 12 |
| **114** | triosephosphate isomerase | gi\|54855 | 27 kDa | 1 |
| **115** | vascular cell adhesion molecule 1, isoform CRA_a | gi\|148680441 (+4) | 81 kDa | 12 |
| **#** | **Identified Proteins: MMP-9 null day 1 only** | **Accession Number** | **Mol. Weight** | **Fraction** |
| **1** | 14-3-3 protein gamma | gi\|9507245 (+1) | 28 kDa | 1 |
| **2** | adiponectin precursor | gi\|26334297 | 27 kDa | 1 |
| **3** | alpha-1-acid glycoprotein 1 precursor | gi\|6679182 | 24 kDa | 1 & 12 |
| **4** | alpha-1-antitrypsin 1-5 precursor | gi\|6678087 (+1) | 46 kDa | 1 |
| **5** | alpha-1 protease inhibitor 2 | gi\|191844 (+1) | 45 kDa | 1 |
| **6** | anti-idiotypic 4C8 immunoglobulin light chain | gi\|60202487 | 11 kDa | 1 |
| **7** | antithrombin-III precursor | gi\|18252782 | 52 kDa | 1 |
| **8** | apolipoprotein A-IV precursor | gi\|110347473 (+4) | 45 kDa | 12 |
| **9** | apolipoprotein E, isoform CRA_f | gi\|148691234 (+6) | 27 kDa | 1 |
| **10** | ceruloplasmin isoform b | gi\|110347564 (+8) | 121 kDa | 1 |
| **11** | chain A, crystal structure monoclonal Fab specific for Shigella flexneri Y LPS | gi\|34811176 | 24 kDa | 1 |
| **12** | chain A, crystal structure of mouse transthyretin | gi\|161172183 | 14 kDa |  |
| **13** | chain A, crystal structure complex Fab fragment of esterolytic Ab | gi\|37926733 | 24 kDa | 1 |
| **14** | chain A, structure for Ab Hyhel-63 Y33a | gi\|75765479 | 24 kDa | 1 |
| **15** | chain C, crystal structure VEGF-B in complex with Ab Fab fragment | gi\|211939056 | 24 kDa | 1 |
| **16** | chain L, anti-carcinoembryonic antigen monoclonal Ab A5b7 | gi\|2194074 | 23 kDa | 1 |
| **17** | chain L, complex of The Neisserial pora P1.4 epitope & 2 Fab-fragments | gi\|73536285 | 23 kDa | 1 |
| **18** | chain L, crystal structure human Il-18 complexed to Ab 125-2h Fab | gi\|241913095 | 24 kDa | 1 |
| **19** | chain L, crystal structure of the antiflavivirus Fab4g2 | gi\|62738079 | 23 kDa | 1 |
| **20** | chain L, crystal structure of Fab fragment mouse anti-human Fas Ab Hfe7a | gi\|18655521 | 24 kDa | 1 |
| **21** | chain L, hyaluronidase in complex with monoclonal Igg Fab fragment | gi\|145579746 (+2) | 24 kDa | 12 |
| **22** | chain L, phosphocholine binding Ig Fab McPC603 | gi\|230159 | 24 kDa | 1 |
| **23** | chain M, idiotype-anti-idiotype Fab complex | gi\|1421601 | 24 kDa | 1 |
| **24** | complement factor I precursor | gi\|110347406 (+2) | 67 kDa | 12 |
| **25** | cytochrome c, somatic | gi\|6681095 (+2) | 12 kDa | 1 |
| **26** | extracellular matrix protein 1 precursor | gi\|170295832 (+6) | 63 kDa | 12 |
| **27** | fetuin | gi\|2546995 (+3) | 37 kDa | 1 |
| **28** | fetuin-B isoform 1 | gi\|10947006 (+4) | 43 kDa | 1 |
| **29** | fibrinogen beta chain precursor | gi\|33859809 | 55 kDa | 1 |
| **30** | ficolin-1 precursor | gi\|6679763 (+1) | 36 kDa | 1 |
| **31** | glycogen phosphorylase, muscle form | gi\|6755256 | 97 kDa | 12 |
| **32** | heavy chain of the monoclonal antibody MST2 | gi\|1617396 | 50 kDa | 1 |
| **33** | hemopexin precursor | gi\|160358829 (+2) | 51 kDa | 1 |
| **34** | hepatocyte growth factor activator | gi\|18044173 (+3) | 71 kDa | 12 |
| **35** | Ig gamma-3 heavy chain precursor | gi\|1304160 (+6) | 52 kDa | 12 |
| **36** | Ighg protein | gi\|62024579 | 52 kDa | 1 |
| **37** | Ig kappa light chain | gi\|12832551 | 23 kDa | 1 |
| **38** | inter alpha-trypsin inhibitor, heavy chain 4, isoform CRA_c | gi\|148692823 (+6) | 103 kDa | 1 |
| **39** | kininogen-1 isoform 2 | gi\|12963497 | 48 kDa | 1 |
| **40** | LDHa protein | gi\|13529599 (+5) | 35 kDa | 1 |
| **41** | leucine-rich alpha-2-glycoprotein | gi\|16418335 | 37 kDa | 1 |
| **42** | L-lactate dehydrogenase B chain | gi\|6678674 | 37 kDa | 1 |
| **43** | malate dehydrogenase, cytoplasmic | gi\|254540027 (+3) | 37 kDa | 1 |
| **44** | Mc5 VLCL [Mus sp.] | gi\|1911624 | 24 kDa | 1 |
| **45** | liver carboxylesterase N | gi\|134034200 (+3) | 61 kDa | 1 |
| **46** | mCG147612, isoform CRA_a | gi\|148686594 (+9) | 50 kDa | 1 |
| **47** | monoclonal antibody kappa light chain | gi\|2906108 (+1) | 14 kDa | 1 |
| **48** | neutrophil gelatinase-associated lipocalin precursor | gi\|34328049 | 23 kDa | 1 |
| **49** | parotid secretory protein | gi\|220939489 (+3) | 22 kDa | 1 |
| **50** | proteasome subunit alpha type-3 | gi\|261824000 (+1) | 28 kDa | 1 |
| **51** | proteoglycan 4 isoform 1 | gi\|161086926 (+2) | 135 kDa | 1 |
| **52** | serine protease inhibitor A3M | gi\|148686846 | 47 kDa | 12 |
| **53** | serine (or cysteine) peptidase inhibitor, clade A, member 1C | gi\|14602605 (+1) | 46 kDa | 1 |
| **54** | serine (or cysteine) peptidase inhibitor, clade F, member 2, isoform CRA_b | gi\|148680863 (+3) | 56 kDa | 1 |
| **55** | serine protease inhibitor A3K precursor | gi\|148747546 | 47 kDa | 1 |
| **56** | serine protease inhibitor A3M precursor | gi\|68053255 | 47 kDa | 1 & 12 |
| **57** | serine protease inhibitor A3N precursor | gi\|130503301 (+1) | 47 kDa | 1 & 12 |
| **58** | serum amyloid A (AA at 131) | gi\|200915 (+2) | 10 kDa | 1 |
| **59** | vitamin D-binding protein | gi\|193446 (+2) | 53 kDa | 1 & 12 |
| **60** | vitronectin, isoform CRA_a | gi\|148683633 (+4) | 50 kDa | 12 |
| **61** | zinc finger protein 608 | gi\|113199757 | 162 kDa | 1 |
